# Antibody- and T Cell-Dependent Responses Elicited by a SARS-CoV-2 Adenoviral-Based Vaccine in a Socially Vulnerable Cohort of Elderly Individuals

**DOI:** 10.3390/vaccines10060937

**Published:** 2022-06-13

**Authors:** Martin Moya, Marcela Marrama, Carina Dorazio, Florencia Veigas, Montana N. Manselle Cocco, Tomas Dalotto Moreno, Gabriel A. Rabinovich, Ariel Aleksandroff

**Affiliations:** 1 City Council of Cordoba, 5000 Cordoba, Argentina; mgmarrama@cordoba.gov.ar (M.M.); caridorazio@hotmail.com (C.D.); aaleksandroff@cordoba.gov.ar (A.A.); 2 Facultad de Ciencias Médicas, Universidad Nacional de Córdoba, 5000 Cordoba, Argentina; 3 Laboratorio de Glicomedicina, Instituto de Biología y Medicina Experimental (IBYME), Consejo Nacional de Investigaciones Científicas y Técnicas (CONICET), C1428 Ciudad de Buenos Aires, Argentina; fveigas@dna.uba.ar (F.V.); mmanselle@dna.uba.ar (M.N.M.C.); tdalotto@ibyme.dna.uba.ar (T.D.M.); gabriel.r@ibyme.conicet.gov.ar (G.A.R.)

**Keywords:** COVID-19, immune function, health disparities, nursing home issues

## Abstract

Background: In spite of compelling evidence demonstrating safety and immunogenicity of adenoviral-based SARS-CoV-2 vaccines in the general population, its effects in socially vulnerable elderly individuals are poorly understood. Here we aimed to investigate the efficacy of two doses of combined vector vaccine, the Gam-COVID-Vac (Sputnik-V vaccine), at 14, 42, and 180 days after immunization, in a nursing home for underprivileged population and homeless individuals. Methods: A phase 3, open-label clinical trial involving administration of two adenoviral vectors (Ad26-Ad5) vaccine, in elderly individuals over the ages of 60 years was performed. SARS-CoV-2 Spike RBD-specific IgG antibodies at days 21-, 42- and 180 post-vaccination was analyzed in sera of individuals receiving two doses of the Sputnik-V vaccine with an interval of 21 days. SARS-CoV-2-specific CD8+ T cell responses, measured by intracellular tumor necrosis factor (TNF) was determined by flow cytometry following antigen-specific cultures. Results: A total of 72 elderly adults with a mean age of 72.6 ± 9.5 years-old was selected after applying the inclusion criteria, all corresponding to an underprivileged population. Two-doses vaccination with Sputnik-V vaccine elicited an antibody-mediated immune response (revealed by quantitative detection of SARS-CoV-2-specific IgG antibodies, CMIA) 70% at day 21, 90% at day 42, and 66.1% at day 180. Fully vaccinated individuals had robust SARS-CoV-2-specific T cell responses, evidenced by TNF production in CD4+ and CD8+ T cells in all time periods analyzed. Conclusion: Six months after receipt of the second dose of the Gam-COVID-Vac vaccine, SARS-CoV-2-specific IgG levels declined substantially among the tested population, whereas CD4+ and CD8+ T-cell-mediated immunity remained at high levels. These data suggest that two doses of combined adenoviral-based vaccine elicits a considerable level of SARS-CoV-2 immune responses in elderly individuals, highlighting its safety and immunogenicity in this highly vulnerable population.

## 1. Introduction

Since the first cases of coronavirus disease 2019 (COVID-19) in Wuhan, China, in December 2019, this disease has spread to millions of individuals worldwide. Severe acute respiratory syndrome coronavirus 2 (SARS-CoV-2) was identified in January, 2020. This virus is highly transmissible between humans and has spread rapidly, causing the COVID-19 pandemic [1,2]. Patients infected with SARS-CoV-2, especially older patients and those with pre-existing respiratory or cardiovascular conditions are at greater risk of developing complications, including severe pneumonia, acute respiratory distress syndrome, multiple organ failure, and in some cases death [3,4]. By 3 March 2022 SARS-CoV-2 had infected more than 440 million people and killed more than 5.9 million worldwide [5].

SARS-CoV-2 elicit detectable antibody and T cell-mediated immune responses [6]. Although previous studies suggested detectable humoral responses at least 4 months after infection [7]; the durability of anti-SARS-CoV-2 IgG antibodies after vaccination without repeated exposure is variable. In contrast, robust and durable T cell mediated memory responses have been documented following natural SARS-CoV-2 infection and immunization with different vaccine platforms, including mRNA-1273, BNT162b2, Ad26.COV2.S, and NVX-CoV2373 that cross-recognize viral variants from Alpha to Omicron [8,9]. Although most studies focused on the safety and immunogenicity of vaccines in the general healthy population, the long-term effects of combined adenoviral-based vaccines in a socially-vulnerable elderly population has not been explored.

We conducted a population-based longitudinal sero-epidemiological study in Cordoba, Argentina, starting in April 2021, and three successive follow-ups in May and September 2021. Vaccine immunogenicity was assessed by analyzing SARS-CoV-2-specific immunoglobulin (Ig) G and SARS-CoV-2-specific T cell-mediated immunity evaluated by intracellular cytokines by flow cytometry.

## 2. Methods

### 2.1. Trial Design and Participants

We initially conducted a Phase 3, open-label clinical trial of two adenovirus vectors (Ad26-Ad5) vaccine, involving participants over the ages of 60 years, for the determination of IgG antibodies for Spike RBD at 21- and 42-days post-vaccination. We subsequently expanded the trial to include testing at 180 days after the first dose of Sputnik-V vaccine.

The trial was conducted at the Padre La Monaca home for senior citizens, an institution dependent of the Secretary of Health of the City Hall of Córdoba, Argentina. Enrolled individuals were in good health and provided written informed consent before undergoing any study procedures. We did not screen for evidence of past or current SARS-CoV-2 infection by testing blood or nasal specimens before enrollment. In other words, we did not test for antibodies or evidence of infection on the same day that the first dose of vaccine was given (baseline), but rather 21 days after the first dose was given, which coincides with the second dose of vaccine. However, in order to overcome this limitation, we determined whether individuals had asymptomatic infection prior to Sputnik-V vaccine administration, from the detection of the rapid test for SARS-CoV-2 nucleoprotein (Abbott Panbio, COVID-19 IgG/IgM), with the understanding that N antibodies (IgG) decrease quickly after natural infection and therefore there is only a limited time window for N antibodies from past illnesses to be detected.

### 2.2. Sputnik-V Vaccine

This vaccine is based on two adenovirus vectors (Ad26-Ad5) expressing the Spike protein.

### 2.3. Study Oversight

The Secretary of Health of the City Hall of Córdoba served as the trial sponsor and made all decisions regarding study design and implementation.

The manuscript was written entirely by the authors, with the first two authors serving as overall lead authors. All authors guarantee the completeness and accuracy of the data and adherence of the study to the protocol. No one who is not an author contributed to the writing of the manuscript.

### 2.4. Trial Procedures

The two adenoviral vectors (Ad26-Ad5) vaccine was administered as a 0.5 mL intramuscular injection into the deltoid on days 1 and 21 of the study; the same dose of the vaccine was administered on both days. Follow up for antibody detection was scheduled 21, 42, and 180 days after the administration of the first dose of vaccine. At 180 days, a sample of EDTA-anticoagulated blood of 24 individuals was taken to study T-cell mediated responses of vaccinated individuals, using the ‘COVID-T Platform [8]’, an optimized strategy to study SARS-CoV-2-specific T cell responses. Purification of peripheral blood mononuclear cells (PBMC) was performed as described [10]. A standard toxicity scale was used to grade adverse events. Local and systemic adverse events were analyzed 7 days after each vaccination dose. Data regarding unsolicited adverse events were collected through day 60. Collection of specimens, as well as monitoring for medically attended adverse events, development of new chronic medical conditions, and serious adverse events, was scheduled to continue through 1 year after the last dose.

### 2.5. Assessment of Antibody Responses

Qualitative (rapid test), semi-quantitative (ELISA), and quantitative (CMIA—chemiluminescence) analysis was used to determine SARS-CoV-2-specific IgG responses recognizing S-2P containing an Asp (D) residue at position 614 to the receptor-binding domain on days 21, 42, and 180.

Asymptomatic infections were assessed using the rapid SARS-CoV-2 nucleoprotein (Abbott Panbio, COVID-19 IgG/IgM) Test. Pooled data were obtained in unidentified format from the nursing home resident health record system and institutional review board approval was obtained.

### 2.6. Assessment of T-Cell Responses by Flow Cytometry

For evaluation of the SARS-CoV-2-specific T cell responses, cryopreserved PBMCs were thawed in complete RPMI 1640 (Serendipia) in the presence of 0.1 mg/mL of DNase I (Roche, Basel, Switzerland) and cultured in the presence of 1 ug/mL SARS-CoV-2-specific peptides pools (Miltenyi, Bergisch Gladbach, Germany) for 6 h. Cultures in the absence of peptides, were used as negative controls and stimulation with phorbol-12-myristate-13-acetate (PMA) and ionomycin was included as positive control. Brefeldin A and Monensin (Biolegend, San Diego, CA, USA) were added to cultures for the last 4 h. Cells were then washed, and surface stained for 25 min at room temperature, fixed with 1% paraformaldehyde (Sigma, St. Louis, MO, USA) for 25 min and intracellularly stained following incubation with permeabilization buffer (BD) for 25 min. All samples were acquired on BD LSR Fortessa^TM^ X-20 and analyzed with FlowJo software. Determination of interferon-γ (IFN-γ), interleukin-2 (IL-2), tumor necrosis factor (TNF), as well as assessment of the frequency of CD154+ cells was assessed in viable cells. Antibodies used in the assay are listed in Table S1.

### 2.7. Statistical Analysis

Safety analyses included all the participants who had received two doses of two adenovirus vectors (Ad26-Ad5) vaccine. The data were extracted from the “Padre la Mónaca” Home for the Elderly and were collected in a Microsoft^®^ Excel spreadsheet. A database was then generated in SPSS, IBM^®^ for statistical analysis.

Numerical variables were presented as means and standard deviations and nominal variables as percentages. Comparisons of numerical variables were performed with the Student’s *t* test for numerical variables and if the distribution was abnormal, the Wilcoxon test was used; nominal variables were analyzed with the Chi-square or Fisher test as appropriate. Correlations between variables and simple and multiple linear regressions were performed; models were tested with the test for comparison of means of related variables. Formulas for predictive models were created.

## 3. Results

### 3.1. Trial Population

A total of 72 elderly adults with a mean age of 72.6 ± 9.5 were eligible after applying the inclusion criteria, all of them corresponding to an underprivileged population. They received the first and second doses of the vaccine with 21 days interval. The demographic characteristics of the participants are shown in Table 1. The studied population had the following general features: 30.1% were women, the mean age of the population was 72.6 ± 9.4 years and the mean body mass index (BMI) was 25.1 ± 7.7, stratified as follows: underweight 23.9%, normal 28.2%, overweight 21.1%, and obese 27.8%. Of the total number of patients, 68.5% were self-supporting or independent and 31.5% were semi-dependent or dependent.

**Figure 1:**
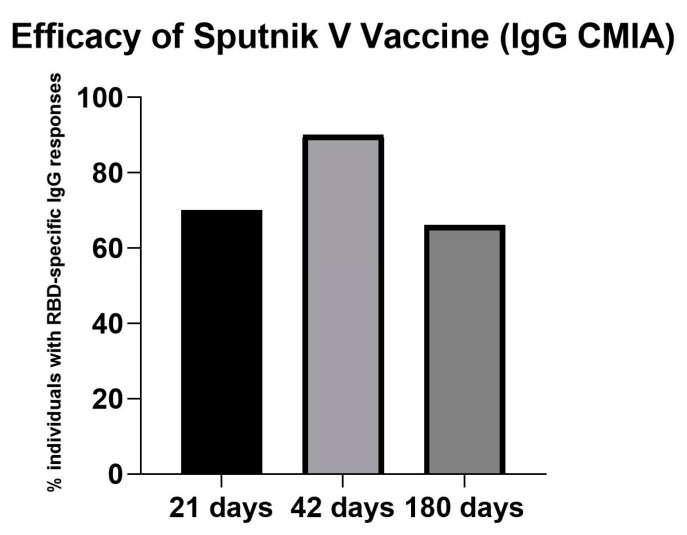
Efficacy of the Sputnik V vaccine as measured by the percentage of individuals displaying RBD-specific IgG responses at days 21, 42, and 180 post-immunizations. These efficacy data were taken only in older adults who had not been previously infected with COVID-19, as detected by SARS-CoV-2 nucleoprotein.

In the cohort analyzed, Sputnik-V vaccine induced a robust immune response (quantitative detection of IgG antibodies against Spike RBD, CMIA) at 21 days in 70%, at 42 days in 90% and at 180 days in 66.1% of the participants (Figure 1).

**Figure 2:**
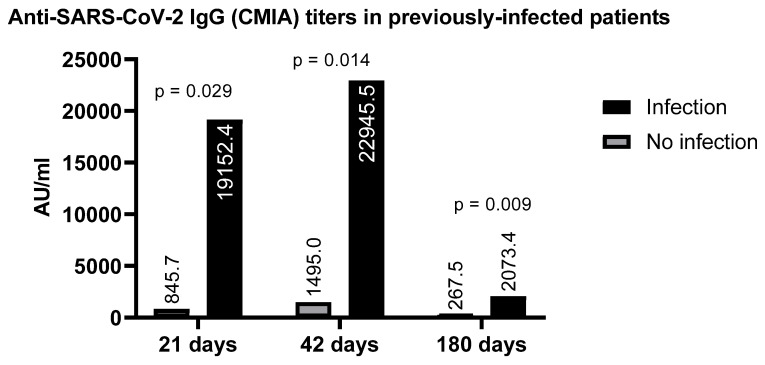
Difference in RBD-specific IgG antibodies in potentially previously infected individuals based on SARS-CoV-2 nucleoprotein detection who were immunized with Sputnik-V vaccine versus those who were SARS-CoV-2 nucleoprotein negative, taken as previously uninfected.

Those individuals who were previously infected (whose sera had antibodies against the nucleoprotein), produced higher levels of Spike RBD-specific IgG upon receiving the vaccine than individuals who had never been in contact with the virus, in spite of receiving full immunization protocol. The frequency of RBD-specific antibodies at 42 days post-vaccination in the previously infected group (individuals who had no symptoms, but asymptomatic infection was detected by the presence of antibodies) was significantly higher (22945.5 AU/mL vs. 1495 AU/mL *p* = 0.014) as compared with patients not previously infected (Figure 2). Likewise, exposure to other viruses, such as hepatitis B (HBV; core positive), was also reflected by an increase in the production of antibodies to SARS-CoV-2 (9487.6 vs. 1832.2; *p* = 0.013), when compared to vaccinated individuals without previous infection.

**Figure 3:**
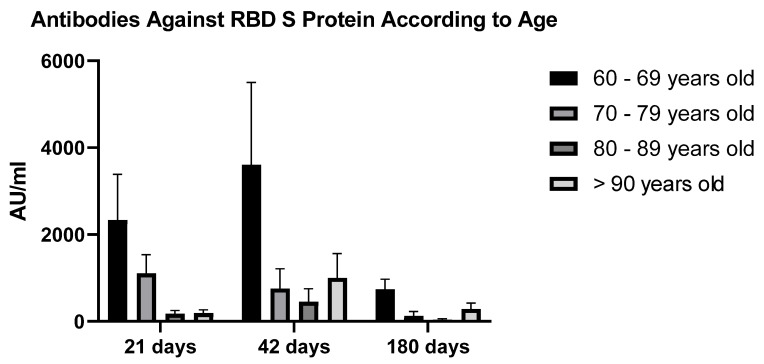
Antibody levels obtained at 21-, 42- and 180-days post-vaccination, following stratification into 4 different groups according to their age (P = NS (Group 60–69 versus 70–79, 80–89, >90 years old at all time periods analyzed) (ANOVA)).

Patients receiving the Sputnik-V vaccine were stratified according to age. Of those aged 61–70 years, 63.6% developed reactive antibodies at 21 days and 87.9% did so at 42 days; while among those over 70 years, reactivity was 66.7% and 83.3%, respectively. Stratification of patients into age ranges of 60–69 years, 70–79 years, 80–89 years, and 90–99 years showed that the 60–69 years group presented higher antibody concentration values, 3078.4 ± 1585.5 AU/mL and 4947 ± 2268.1 AU/mL for measurements taken on days 21 and 42, respectively (Figure 3).

**Figure 4:**
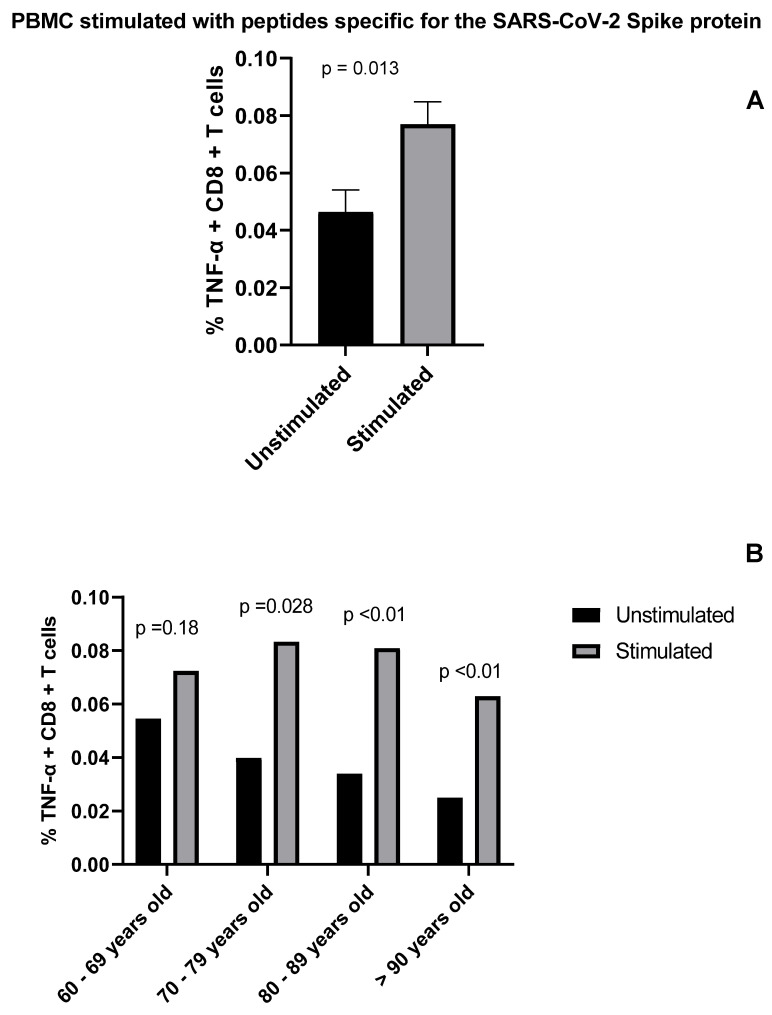
Determination of SARS-CoV-2-specific CD8^+^ T cell responses in Sputnik-V vaccinated individuals. PBMCs of individuals were stimulated with SARS-CoV-2-specific peptide pools. Percentage of TNF −α^+^ cells gated on CD8^+^ T cells was determined by flow cytometry. (**A**) Bar graph show frequency of CD8^+^ TNF −α^+^ cells. (**B**) The graph shows the percentage of CD8^+^ TNF −α^+^ cells following age group stratification.

T cell-mediated immunity plays a central role in the control of SARS-CoV-2 infection and is a key component of immunization strategies. Particularly, both CD4+ and CD8+ T cells have been reported to control multiple viral infections and provide protection against subsequent re-infections by generating immunological memory [11]. Previous studies have shown that both convalescent COVID-19 patients and individuals vaccinated with any of the different COVID-19 vaccine platforms develop long-term immunity mediated by CD4+ and CD8+ specific T cells [11]. We analyzed SARS-CoV-2 specific T cell responses at 180 days post-vaccination in our cohort as measured by cytokine production after stimulation with peptide pools that cover the immunodominant sites of SARS-CoV-2 Spike protein. We found that fully vaccinated individuals had robust specific T cell responses as shown by the increased percentage (2-fold) of cells positive for TNF compared to the unstimulated controls (Figure 4). Moreover, we also found a higher percentage of IFN-γ+ cells when comparing stimulated vs. unstimulated samples (data not shown). A similar outcome was observed when IL-2 was evaluated (data not shown).

### 3.2. Vaccine Safety

No serious adverse events were reported, and no pre-specified trial-halting rules were met in any of the individuals analyzed. The most commonly solicited adverse events were headache, fatigue, and injection-site pain. Local events were more common after the administration of the second dose of the vaccine. These symptoms typically occurred on the day of vaccination or 1 day afterward and resolved soon. Those patients who had nonspecific symptoms were treated with ibuprofen or acetaminophen.

## 4. Discussion

On 2 February 2021, the County Government of the city of Cordoba (Argentina) suggested that the elderly residents of the long-term care facility (Padre Lamónaca) will receive the Sputnik-V vaccine. In this study we determined the levels of IgG antibodies at days 21, 42 and 180 after the first dose in individuals who had not been exposed to SARS-CoV-2 and who received the 2 doses 3 weeks apart.

On the basis of published results from vaccine trials and other data sources, it is estimated that people immunized against SARS-CoV-2 would experience a decline in approximately half of their protective antibodies every 108 days or so. As a result, vaccines that initially offered 90% protection against mild cases of disease might only be 70% effective after 6 or 7 months [12]. In fact, immunological studies have documented a steady decline of antibody levels among vaccinated individuals [13]. Long-term follow-up of vaccine trial participants has revealed a growing risk of breakthrough infection [14]. Health-care records from countries such as Israel, the United Kingdom, and other countries all show that COVID-19 vaccines lose their potency over time. In our study we observed a similar behavior in the case of humoral immunity, where antibodies titers increased progressively until 42 days post-vaccination, and then decreased to values below those reached at 21 days post-vaccination. A possible explanation for the observed decrease in the production of antibodies stimulated by the Sputnik-V vaccine, in comparison to the work published previously [15], include the study of a population of older adults (mean 72.6 ± 9.5; minimum 61–maximum 97), history of malnutrition in the population studied (23.9% of older adults were underweight and 48.9% were overweight), and the fact that all these individuals belong to a vulnerable population with a very low socioeconomic level, many of them being homeless.

The antibody concentration values obtained 14 days after the second dose of the vaccine are promising. However, these values also highlight the importance of monitoring antibody responses post-vaccine administration, especially among the elderly socially-vulnerable population, who could potentially be immunocompromised.

In addition to humoral responses, understanding the nature and magnitude of SARS-CoV-2-specific T cell responses is essential to monitor vaccine effectiveness. In other coronaviruses of the family (i.e., SARS-CoV-1), antibody levels fall below the detection limit between 1 and 3 years [16], while memory T lymphocytes remain active up to 11 years later [17]. At the same time, recent studies in patients recovered from COVID-19, revealed the fundamental value of T lymphocytes (both CD4+ and CD8+ T cells) [11] in conferring protection against SARS-CoV-2. In order to monitor the immunological memory elicited by vaccination, we measured the antigen-specific T cell responses. Individuals involved in this study exhibited a robust T cell response against SARS-CoV-2 peptides. Circulating memory T cells elicited by Sputnik-V vaccine in elderly individuals produced high amounts of TNF and IL-2 following stimulation with SARS-CoV-2 Spike-derived peptides. Particularly, statistically significant differences were observed for TNF-producing CD8+ T cells with respect to unstimulated cells.

Neutralizing antibodies that can intercept viruses before they infect cells might not be protective during the whole infection cycle. Although antibody levels typically rise after vaccination, they rapidly decline months later. In contrast, cellular responses are longer lasting. Memory B cells, which can rapidly deploy more antibodies in the event of re-exposure to the virus, tend to stay on-site, and so do memory CD8+ T cells, which can exert cytotoxic activity toward infected target cells. Both memory cell populations provide a critical protection in cases of SARS-CoV-2 infection. In fact, vaccination stimulated long-lasting responses when both arms of adaptive immunity were considered simultaneously [18]. Memory B cells continued to grow in number for at least six months, and enhanced their ability to fight the virus over time. On the other hand, T cell counts remained relatively stable, decreasing only slightly during the study period [18].

In this regard, analysis of lymph nodes from vaccinated individuals revealed the appearance of germinal centers that produced increasingly potent activated follicular B cells over time [19]. The B cells in these structures randomly mutated their genes (somatic hypermutation) to create an entirely new set of antibodies. Cells that produced the best antibody repertoires eventually prevailed through an evolutionary process that enhanced the immune system’s ability to fight other SARS-CoV-2 variants [8,9]. These germinal centers persisted for 15 weeks after immunization with an RNA platform-based vaccine, a response which was much longer than those previously seen with older technology vaccines for other diseases. Our work shows that vaccinated individuals, with only two doses of a combined adenoviral vaccine, display robust T cell-mediated responses and do not acquire moderate or severe COVID-19 infections, highlighting the safety and immunogenicity of this vaccination strategy in socially vulnerable elderly individuals.

Lowering infection rates should help break the cycle of viral transmission, ultimately resulting in fewer cases of severe COVID-19 infection and reduced death rates, thus keeping the emergence of vaccine-resistant variants at bay. Resistant viruses are more likely to emerge when transmission is not controlled [20]. Getting more people vaccinated and protecting the most vulnerable population is the most effective intervention to keep transmission rates low.

## 5. Conclusions

This work reaffirms the efficacy of the Sputnik-V vaccine, showing high levels of immunization, even in an elderly, underprivileged population with very low resources. We also found that immunity decreases as the age of the vaccinated individuals increases. Finally, we found that cellular immunity persists even after humoral immunity declines, suggesting that immunized older adults, as well as the general healthy population, are still protected even if antibodies decline.

## Figures and Tables

**Table 1:** Characteristics of the participants at baseline.

Characteristic	All Participants (N = 72)
Sex—no. (%) Male Female	51 (69.9) 22 (30.1)
Age—year *	72.6 ± 9.4
Body-mass index (BMI) *ˆ	25.1 ± 7.7
BMI Category—no. (%) Underweight Normal range Overweight Obese (Class I) Obese (Class II) Obese (Class III)	17 (23.9) 20 (28.2) 15 (21.1)4 (5.6) 4 (5.6)
Type of patient Ambulatory Bedridden	50 (68.5) 23 (31.5)

* Plus-minus values are means ± SD. ˆ The body-mass index is the weight in kilograms divided by the square of the height in meters.

## Data Availability

The data and materials are open to all health agents who wish to view and analyze them.

## Supplementary Materials

The following supporting information can be downloaded at: https://www.mdpi.com/article/10.3390/vaccines10060937/s1. Table S1: Antibodies used to assess SARS-CoV-2-specific T cell-mediated immunity.
