# Supplementary material for: Antibody- and T Cell-Dependent Responses Elicited by a SARS-CoV-2 Adenoviral-Based Vaccine in a Socially Vulnerable Cohort of Elderly Individuals
Source: Vaccines (Basel). 2022 Jun 13;10(6):937. doi: 10.3390/vaccines10060937 (PMC9228665; doi:10.3390/vaccines10060937)
Supplement: Supplementary file 1 [file vaccines-10-00937-s001.zip › vaccines-1720932-supplementary.pdf]

Supplementary Material

Table S1. Antibodies used to assess SARS-CoV-2-specific T cell-mediated immunity.

**Antibody**	**Fluorochrome**	**Clone/vendor/catalogue**
CD3	BV510	OKT3/Biolegend/317332
CD4	APCCy7	OKT4/Biolegend/317418
CD8	PE	RPA-T8/Biolegend/301064
CD154	FITC	24-31/Biolegend/310804
IFN- γ	PerCP-Cy5.5	4S.B3/Biolegend/505822
TNF- α	PECy7	MAb11/Biolegend/502930
IL-2	APC	MQ1-17H12/Biolegend/500310
